# S737F is a new CFTR mutation typical of patients originally from the Tuscany region in Italy

**DOI:** 10.1186/s13052-017-0443-z

**Published:** 2018-01-03

**Authors:** Vito Terlizzi, Antonella Miriam Di Lullo, Marika Comegna, Claudia Centrone, Elisabetta Pelo, Giuseppe Castaldo, Valeria Raia, Cesare Braggion

**Affiliations:** 1 Centro Regionale Toscano per la Fibrosi Cistica, Azienda Ospedaliero-Universitaria Meyer, Via Gaetano Pieraccini 24, 50141 Florence, Italy; 2 CEINGE-Biotecnologie Avanzate scarl, Naples, Italy; 3 Dipartimento di Medicina Molecolare e Biotecnologie Mediche, Università di Napoli Federico II, Naples, Italy; 4 Dipartimento di Neuroscienze, Sezione di ORL, Università di Napoli Federico II, Naples, Italy; 5 SOD Diagnostica Genetica, Azienda Ospedaliera Universitaria Careggi, Florence, Italy; 6 Centro Regionale Fibrosi Cistica Unità Pediatrica, Dipartimento di Scienze Mediche Traslazionali, Università di Napoli Federico II, Naples, Italy

**Keywords:** Cystic fibrosis, CFTR, Nasal brushing, Functional analysis, Gating, Genotype-phenotype correlation, Tuscany region, CFSPID, CRMS

## Abstract

**Background:**

An increasing number of patients have been described as having a number of *Cystic Fibrosis Transmembrane conductance Regulator* (CFTR) variants for which it lacks a clear genotype–phenotype correlation. We assesses the clinical features of patients bearing the S737F (p.Ser737Phe) CFTR missense variant and evaluated the residual function of CFTR protein on nasal epithelial cells (NEC).

**Methods:**

A retrospective database was performed from individuals homozygous or compound heterozygous for the S737F variant followed in the Cystic Fibrosis (CF) Centre of Florence. We performed a nasal brushing in cooperating patients and compared the results with those of patients followed in the pediatric CF Centre of Naples.

**Results:**

9/295 (3%) subjects carrying at least S737F CFTR variant on one allele were identified. Patients were diagnosed in 7/9 cases by newborn screening and in two cases for dehydration with hypochloremic metabolic alkalosis; at diagnosis sweat chloride levels (SCL) were in the pathological range in only one case. After a mean follow up of 8,6 years (range 0,5–15,8), SCL were in the pathological range in 8/9 cases (mean age at CF diagnosis: 1,5 years), all patients were pancreatic sufficiency and respiratory function was normal. The gating activity on NEC was 15.6% and 12.7% in two patients compound heterozygous for W1282X and DelE22_24, while it was ranged between 6,2% and 9,8% in CF patients.

**Conclusions:**

S737F is a CFTR mutation associated to hypochloremic alkalosis in childhood, mild CF phenotype in teenage years and a residual function of CFTR protein.

## Background

Cystic fibrosis (CF) is the most common life-threatening autosomal recessive disease in the US, affecting approximately 1 in 4000 newborns [1]. The prevalence in Europe is 0.737 per 10,000 and the incidence in Italy is estimated at 1/4,238 live [2]. It is caused by mutations of the CF *Transmembrane conductance Regulator* gene (*CFTR*) that encodes the cAMP-regulated chloride channel. About 2000 variants were described in the *CFTR* gene so far [3]. CFTR mutations can be classified into 6 classes according to the mechanism by which they impair CFTR protein synthesis, trafficking or function: class I mutations cause absent CFTR production, class II mutations affect intracellular processing of CFTR and class III mutations lead to defective regulation of the CFTR channel at the apical plasma membrane; classes IV, V and VI are associated to some degree of residual function [3, 4].

Although CFTR functions mainly as a chloride channel, it has many other regulatory roles, including inhibition of sodium transport through the epithelial sodium channel, regulation of the outwardly rectifying chloride channel, regulation of ATP channels, regulation of intracellular vesicle transport, acidification of intracellular organelles, and inhibition of endogenous calcium-activated chloride channels. CFTR is also involved in bicarbonate–chloride exchange. There are several hypotheses regarding how CFTR dysfunction leads to the phenotypic disease. One of the major is the low-volume hypothesis postulating that the loss of inhibition of epithelial sodium channels, because of CFTR dysfunction, leads to excess sodium and water reabsorption, resulting in dehydration of airway surface materials [5].

Newborn screening (NBS) offers the opportunity for early diagnosis and improved outcome in patients with CF [6–8]. There continues to be considerable variability in structure of NBS programs [9]. NBS is done by the measurement of immunoreactive trypsinogen (IRT) in blood spots taken from newborn infants at day 3. Infants who have a high IRT concentration (>99th centile) undergo further assessment via a repeat IRT, 3 weeks later (IRT/IRT2), or by analysis of the initial blood spot for a specified group of CFTR mutations (IRT/DNA). A sweat test must still be done to confirm the diagnosis in infants with a positive NBS result. The sweat test remains the most readily available and clinically useful way of making the diagnosis of CF, provided it is done according to strict guidelines, with pilocarpine iontophoresis and a quantitative determination of chloride concentration [1, 10, 11]. A diagnosis of CF can be made in a patient with a positive NBS result and a sweat chloride concentrations greater than 60 mmol/L [1, 10].

The CF phenotype includes multi-organ involvement with predominant severe respiratory disease, pancreatic insufficiency (PI) and male infertility [5]. In the last years an increasing number of patients have been described as having CFTR-related disorder (CFTR-RD) [12]. These cases are generally characterized by a later onset of symptoms such as pancreatitis, [13] disseminated bronchiectasis, congenital bilateral absence of vas deferens (CBAVD) associated to normal or borderline sweat chloride concentrations (30–59 mmol/L for infants less than 6 months of age, 40–59 mmol/L for older individuals) and two CF mutations with a different degree of CFTR protein dysfunction [14]. Finally, the growing proportion of NBS programs revealed a large number of infants having a Cystic Fibrosis Screen Positive, Inconclusive Diagnosis (CFSPID) in Europe or CFTR – related metabolic syndrome (CRMS) in the US including those infants with elevated IRT levels, but with insufficient sweat chloride or genetic data to support a diagnosis of CF [15, 16].

Nowadays the use of CFTR gene sequencing [17] led to the detection of a number of variants for which it lacks a clear genotype–phenotype correlation. Some of these can reach a higher frequency only in certain populations, due to a founder effect in ethnic or geographical isolates [18, 19].

S737F (c.2210C > T; p.Ser737Phe) is a CFTR missense variant, located in exon 13 and characterized by a substitution of serine with phenylalanine in position 737, identified in 2004 in two Italian patients with pancreatic sufficiency (PS) and pathological sweat chloride level (SCL) in one case (Cl 71 mEq/l) and borderline in the other (Cl 51 mEq/l) (submitted to http://www.genet.sickkids.on.ca/cftr/app). Functional classification of this variant still remains unknown and no data are available in literature at the moment.

The aim of our study is to describe clinical features of patients bearing such rare variant and to assess the residual function of CFTR protein on nasal epithelial cells obtained by nasal brushing.

## Methods

### Patients population

We performed a retrospective analysis of clinical records of all patients either homozygous or compound-heterozygous for the S737F CFTR missense variant between 295 patients followed in the Cystic Fibrosis Centre of Tuscany region (Italy). Demographic, clinical, genetic and biochemical data regarding all enrolled patients were extracted from local electronic health records. All patients had given consent to the recording of their clinical data and for their anonymous use for scientific purposes, including descriptive studies. Furthermore, the approval of Ethics Committee of University Federico II of Naples was obtained for sampling of nasal epithelial cells and for the functional analysis of novel mutations in patients with CF.

### Diagnostic tests and clinical data

**Fig. 1:**
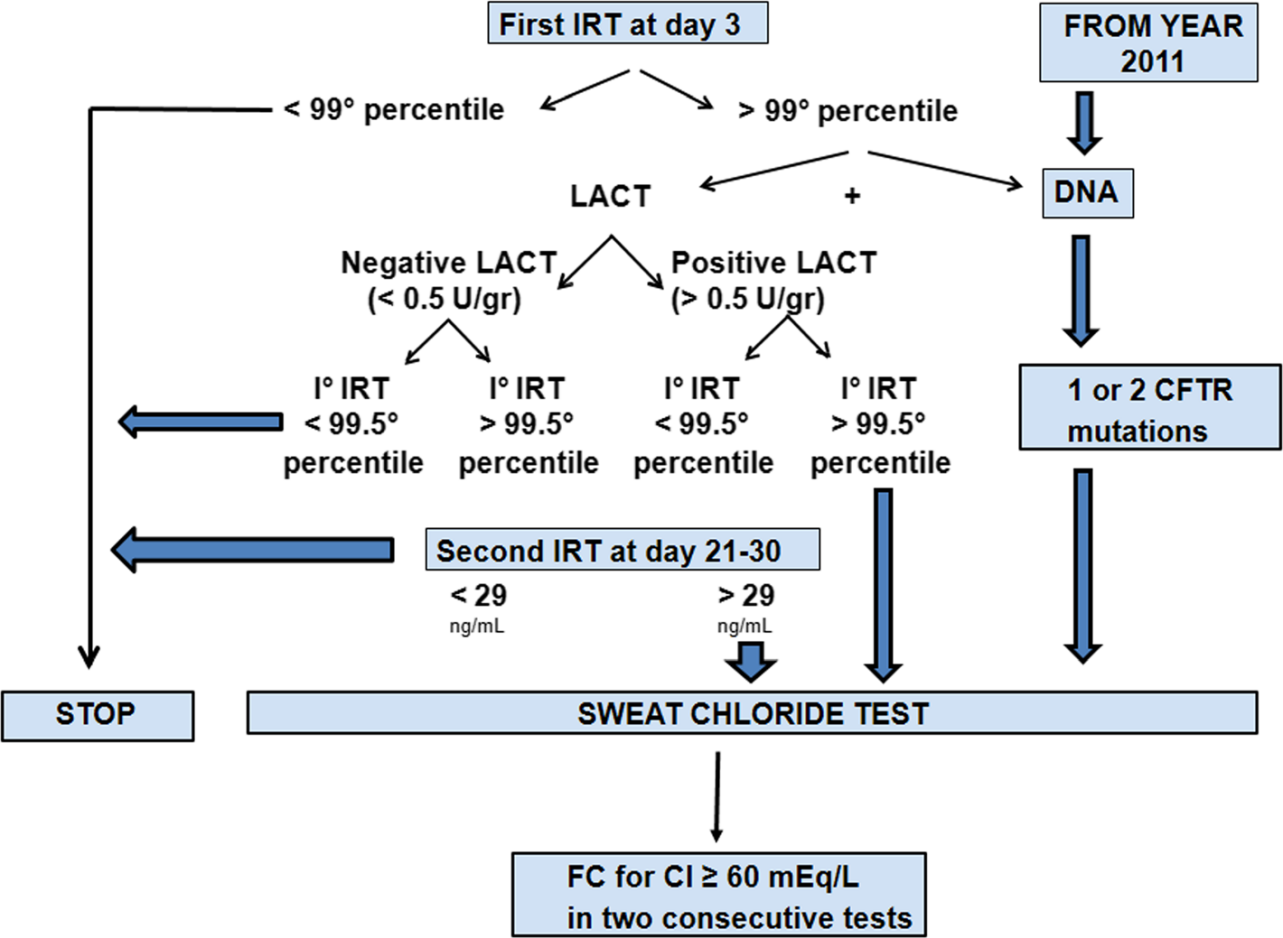
The newborn screening algorithm used at the CF Centre of Florence, Italy

All patients underwent NBS for CF by the analysis of blood IRT at day 3 performed by using Auto-DELFIA method until 2013 and by the GSP instrument from 2014 (Perkin-Elmer). Meconium lactase was detected by glucose production after incubation of meconium with lactose. The NBS algorithm is reproduced in Fig. 1. From 2011 a pilot project was aimed to compare usual diagnostic algorithm (IRT - meconium lactase - IRT2) to a new protocol IRT-DNA.

We collected a new database including demographic data and detailed clinical or genetic data at diagnosis, as follows: gender, age at diagnosis, genotype and cause of diagnosis.

SCL, obtained according to guidelines by the Gibson and Cooke method, [11, 20] were analyzed at diagnosis and annually repeated in each patient with quantitative pilocarpine iontophoresis. Furthermore sweat test was performed in the same laboratory, thus ruling out the lack of harmonization between different labs [21].

We defined CFSPID infants who are asymptomatic, with hypertrypsinogenemia at birth and either: (1) 0 or 1 CFTR mutations, plus intermediate sweat chloride (30–59 mmol/L); or (2) 2 CFTR mutations, at least 1 of which has unclear phenotypic consequences, plus a normal sweat chloride (<30 mmol/L) [15].

Weight/Height pc or body mass index (BMI) pc were evaluated in patients respectively under or beyond 2 years of age [22]. Last best forced expiratory volume in the 1st second (FEV_1_), expressed as percentage of predicted value for age, according to standardized reference equations for spirometry [23] were recorded for patients aged over 6 years. Given the inter-individual variability of FEV_1_ and the evolution of lung damage with age, the patients were classified as “severe” or “mild” according with Schluchter et al. criteria that takes into account both the FEV_1_ value and age [24]. Endobronchial bacterial infections were detected by sputum or throat swab cultures. Pseudomonas aeruginosa (PA), Stenotrophomonas malthophilia (SM), Haemophlus Influenzae (HI), Methicillin sensitive *Staphylococcus aureus* (MSSA) or Methicillin resistant *Staphylococcus aureus* (MRSA) chronic infection was defined following modified Leeds criteria [25]. PS was defined on the basis of at least two values of fecal pancreatic elastase higher than 200 μg/g measured outside acute gastrointestinal diseases [26]. Pancreatitis was defined according to the report from the international study group of pediatric pancreatitis [27]. Cystic fibrosis related diabetes mellitus (CFRD) was diagnosed according to the American Diabetes Association criteria [28]. CF-associated liver disease (CFLD) was defined when imaging (ultrasound, computed tomography, magnetic resonance) and demonstrated hepatic parenchymal abnormalities and/or portal hypertension (esophageal varices, portal-systemic collaterals, splenomegaly) in the absence of other causes [29]. Complications such as allergic bronchopulmonary aspergillosis (ABPA), meconium ileus (MI) or distal intestinal obstruction syndrome (DIOS), nasal polyposis, hemoptysis, and pneumothorax were registered.

### Genetic analysis

According to European recommendations, [30] molecular analysis was performed testing a panel of the most frequent CFTR mutations followed by the search of the most frequent CFTR rearrangements [31] and sequencing of the whole coding regions of CFTR [14] once one or both mutations had not been identified. Before 2011 we performed genetic analysis in all infants with a pathological sweat chloride in order to confirm CF diagnosis, while since 2011 it has become part of the screening program (IRT-DNA) (Fig. 1).

### Nasal brushing

In order to assess the residual function of CFTR protein, we performed a nasal brushing in cooperating patients bearing S737F CFTR variant and compared the results with those of CF patients with other genotypes followed in the pediatric Cystic Fibrosis Centre of Campania region (Italy). The informed consent was required to the patients (legal guardian for minors) before sampling, after a complete description of the aims of the study. All subjects underwent a complete ear-nose-throat evaluation. To test the activity of the CFTR protein, we used the halide-sensitive fluorescent system. Details are provided in a previous paper of our group [32, 33].

## Results

### Genotype, clinical data and clinical course

Nine subjects carrying at least one S737F were identified after gene sequencing (detection rate 98%): 8 were compound heterozygous for the S737F and one class I–II mutation (mean age: 8,9 years, range 0,5–15,8 years), while one patient (8,4 years) was homozygous for the S737F (Table 1).

**Table 1:** demographic data of the enrolled patients

Patient	Gender	Age at diagnosis (months)	Cause of diagnosis	Second mutation	SCL at diagnosis	IRT^a^ (ng/ml)
1	M	1	NBS	F508del	46	74
2	M	20	Hypochloremic Alkalosis	S737F	45	31
3	M	1	NBS	F508del	35	76
4	F	1	NBS	541delC	48	108
5	M	10	Hypochloremic Alkalosis	22,23,24 del	71	37
6	M	1	NBS	W1282X	51	79
7	F	1	NBS	F508del	52	100
8	F	1	NBS	F508del	49	76
9	M	1	NBS	F508del	51	76

*SCL* sweat chloride level, *IRT* blood immunoreactive trypsinogen, *NBS* newborn screening

^a^IRT is elevated for values >99th centile, variable every 3–4 months from 50 to 64 ng/ml

No detection of complex alleles has been found. 7/9 patients were diagnosed by NBS and were considered CFSPID infants having a borderline SCL and one CFTR causing mutation. In the remaining two cases (resulted negative at NBS) diagnosis was performed at the age of 10 months and 20 months respectively, after an episode of dehydration with hypochloremic metabolic alkalosis during the summer needing hospitalization. At diagnosis, one case had SCL in the pathological range (Cl: 71 mEq/L) while in 8/9 cases SCL was in the intermediate range for age (Cl 30–59 mEq/L for infants under the age of 6 months; Cl 40–59 mEq/L for patients over 6 months) (Table 1) [10]. During follow up SCL, annually o semesterly evaluated in each patient, gradually increased with age: the last SCL was pathological for age (range 60–121 mEq/L) in 8/9 patients (defining a CF diagnosis), while in only one case it remains unmodified in borderline range (Table 2). The mean age at diagnosis of CF was 1,5 years (range 0,2–3,8).

**Table 2:** Clinical data of the enrolled patients

Patient	Age (years)	PS	FEV_1_	Sputum pathogens	Pc BMI (°)	SCL at enrolment (mEq/l)	Follow up (years)
1	9,5	Yes	NA	Hi, Mssa	95	46	9,5
2	8,4	Yes	99	Mssa	>95	98	6,6
3	9,7	Yes	101	Hi	95	78	9,7
4	8,8	Yes	96	Mssa, Sm	75–85	94	8,8
5	15,7	Yes	99	Pa	75–85	89	14.9
6	15,8	Yes	108	Mssa	50–75	121	15,8
7	10,7	Yes	114	Mssa	> 95	60	10,7
8	0,5	Yes	NA	Mssa	>95^a^	68	0,5
9	1,5	Yes	NA	Hi	90^a^	67	1,5

*PS* pancreatic sufficiency, *FEV*_*1*_ forced expiratory volume in the 1st second, *SCL* sweat chloride level, *NA* not available, *Hi* Haemophilus Influenzae, *Mssa* Methicillin sensitive *Staphylococcus aureus*, *Sm* Stenotrophomonas malthophilia, *Pa* Pseudomonas Aeruginosa

^a^Weight/Height pc were evaluated in patients under 2 years of age

Lung function tests were available in 6 cooperating patients: according to Schluchter et al. criteria, [24] all patients were classified as “mild” for age (Table 2).

After a mean follow up of 8,6 years (range 0,5–15,8), all patients were PS and in good nutritional status (with five of them having a BMI ≥ 95° pc). None of them had presented pancreatitis, CFLD, CFRD or other complications. Sputum or throat swab cultures revealed chronic infection by MSSA in 6/9 cases and occasional infection of PA, SM, or HI in the other cases (Table 2).

Respiratory exacerbations, requiring oral antibiotics prescription were uncommon in all patients over the time (only the patient n.5 required a cycle of oral ciprofloxacin and aerosol therapy in order to eradicate PA infection). Chest High Resolution Computed Tomography scan showed the absence of bronchiectasis in patients n. 5 and n. 6. In order to exclude CBAVD presence, we proposed the analysis of spermiograms for the two males adolescents patients but the parents refused.

### Nasal brushing

**Fig. 2:**
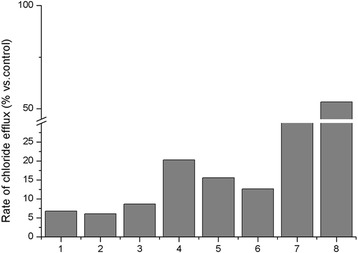
CFTR gating activity on nasal epithelial cells obtained from CF patients and carriers with different genotypes. 1: F508del/F508del; 2: F508del/[R74;V201 M;D1270]; 3: N1303 K/711 + 1G > T; 4: W1282X/D1152H; 5: S737F/W1282X; 6: S737F/DelE22_24; 7: F508del (heterozygous); 8: G542X (heterozygous)

As shown in Fig. 2, we analyzed the CFTR gating activity on nasal epithelial cells obtained from several CF patients and carriers. In particular, we studied: i) three patients with CF of which one was homozygous for the F508del mutation (case #1), another was compound heterozygous for the N1303 K and the [R74;V201 M;D1270] complex allele (case #2) and the third was compound heterozygous for the N1303 K and the 711 + 1G > T mutations (case #3) (the phenotypes are showed in Table 3) [32]. They showed an activity of 6,2%, 9,8% and 5,2%, respectively; ii) a CF patient compound heterozygous for the W1282X and D1152H mutation [32] (Table 3). He had an activity of 20.3% (case #4); iii) two patients compound heterozygous for the S737F variant with the W1282X mutation (case #5) and with the DelE22_24 (case #6). Such cases showed an activity of 15.6% and 12.7%, respectively (the phenotypes are showed in Table 2 and in Table 3, cases # 5 and 6); iv) two carrier subjects, heterozygous for the F508del mutation (case #7) and for the G542X mutation (case #8); they had an activity of 40.7 and 76,8% (Table 3).

**Table 3:** Clinical data and CFTR gating activity on nasal epithelial cells obtained from several CF patients and carriers

Patient	Age (years)	Genoytpe	SCL at diagnosis (mEq/L)	PS	FEV_1_ at enrollment (%)	Sputum Pathogens	CFTR gating (%)
Case #1	19	F508del/F508del	118	NO	64	Pa	6,2
Case #2	21	N1303 K/ [R74;V201 M;D1270]	109	YES	119	Hi, Mssa	9,8
Case #3	23	N1303 K/711 + 1G > T	89	NO	54	Pa	5,2
Case #4	19	W1282X/D1152H	52	YES	115	Mssa	20,3
Case #5	15,7	S737F/W1282X	71	YES	99	Pa	15,6
Case #6	15,8	S737F/Dele22_24	51	YES	108	Mssa	12,7
Case #7	42	F508del heterozygous	NA	NA	NA	NA	40,7
Case #8	48	G542X heterozygous	NA	NA	NA	NA	76,8

*PS* pancreatic sufficiency, *FEV*_*1*_ forced expiratory volume in the 1st second, *SCL* sweat chloride level, *NA* not available, *Hi* Haemophilus Influenzae, *Mssa* Methicillin sensitive *Staphylococcus aureus*, Pa Pseudomonas Aeruginosa

## Discussion

We described the first cohort of patients bearing the S737F CFTR missense variant and evaluated the residual function of CFTR protein on nasal epithelial cells obtained by nasal brushing in comparison to patients with other genotypes. Our results show that S737F is associated to hypochloremic alkalosis in childhood, mild CF phenotype in teenage years and a residual function of CFTR protein. In fact, despite 7 patients resulted positive to NBS, the other two were diagnosed by a single episode of dehydration with hypochloremic metabolic alkalosis. Furthermore, all 9 patients presented PS and good nutritional status, all had normal respiratory function for age and none of them developed complications during follow up. Only one patient presented an occasional isolation of *P. aeruginosa* that eradicated after antibiotic therapy. Nevertheless the mean age and the follow up of our patients are too low to exclude a severe course of the pulmonary disease in adulthood or the development of pancreatic insufficiency.

It is known that some mutations are more frequent in specific regions or ethnic groups [18, 19]. Interestingly all the families of the patients were residing in the Tuscany region for at least two generations. Our data confirm the need to investigate the regional origin of each patient with suspected CF in order to reduce the residual risk that a couple negative for the test will have an affected child [19]. Furthermore is also necessary to adapt neonatal screening panels based on the frequency of mutations in each region.

The management of children with an inconclusive diagnosis following NBS has been recently discussed [15, 16]. In some cases the sweat chloride result may be in intermediate range or CFTR gene changes may be recognized, the phenotypic consequences of which are unclear. According to Munck et al. and Ooi et al., in our cohort we repeated annually or semesterly the sweat test and during follow up 6/7 CFSPID infants moved to a CF diagnosis given that sweat test moved from an intermediate to a CF-confirmatory result [6, 33, 34]. Unlike Ooi et al. [33] sweat chloride increased to abnormal levels at a slightly lower mean age (21,3 months vs 17 months).

The sensitivity of NBS for CF diagnosis depends on the IRT cut off as well as the population screened as a result of differences in the genotypic distribution for CF in the population, with estimates of false negative results ranging from 1.7–5.4% of children with CF [35, 36]. Delay in diagnosis of CF and, hence, delay in treatment is associated with deleterious outcomes including nutritional and growth concerns, a higher rate of complications, increased stress to the families, and the potential for poorer pulmonary outcomes [37–39]. Nevertheless, the effect of age of diagnosis on survival with CF is moot given that it is not the only factor affecting the survival [36, 40–42]. In a 30 year retrospective review of survival with CF from two regions in Italy including diagnosis by symptoms and NBS, Assael et al. reported that patients diagnosed after 5 years of age appeared to have a survival advantage over those diagnosed under 1 year of age without a significant effect of NBS on survival [40].

In our cohort two patients (cases 2 and 5) resulted negative at NBS and were diagnosed at the age of 10 months and 20 months after an episode of dehydration with hypochloremic metabolic alkalosis during the summer. This is a relatively common and sometimes dangerous manifestation of CF in infancy; the possibility of CF diagnosis should be considered in any infant with this metabolic disorder even if resulted negative at newborn screening. In order to prevent these events we have included S737F mutation in cystic fibrosis neonatal panels screening and we suggest to look for S737F in subjects of Tuscany region origin with symptoms compatible with cystic fibrosis.

The analysis of CFTR gating activity on ENC confirms that such analysis may help to define the residual activity of the mutated protein and seems to correlate with the severity of the CF phenotype. In fact, the three patients with severe CF and compound heterozygous for two *CFTR* severe mutations (cases 1 to 3) show a very low residual CFTR activity (i.e., < 10%); the two heterozygous subjects show an activity between 40 and 80%, and the patient with mild CF, compound heterozygous for the W1282X and the mild D1152H mutation [43] had an activity in the range 10 to 20%. The two cooperating patients bearing the S737F mutation show a residual CFTR activity of 12.7 and 15.6%, according to a mild CF clinical expression (that seems to be associated to residual activity ranging from 10 to 20%).

## Conclusions

S737F seems to be a CFTR mutation, typical of patients originally from the Tuscany region, associated to a residual activity of the CFTR protein assessed in nasal epithelial cells. We have included this mutation in cystic fibrosis neonatal panels screening in order to prevent dangerous episodes of dehydration with hypochloremic metabolic alkalosis.

## Abbreviations

ABPA: Allergic bronchopulmonary aspergillosis
BMI: Body mass index
CF: Cystic fibrosis
CFLD: CF liver disease
CFRD: CF related diabetes
CFSPID: Cystic Fibrosis Screen Positive, Inconclusive Diagnosis
CFTR: Cystic fibrosis transmembrane regulator
CFTR-RD: CFTR-related disorder
CRMS: CFTR-Related Metabolic Syndrome
DIOS: Distal intestine obstructive syndrome
HI: Haemophilus Influenzae
IRT: Immunoreactive trypsinogen
MI: Meconium ileus
MRSA: Methicillin resistant *Staphylococcus aureus*
MSSA: Methicillin sensitive *Staphylococcus aureus*
NBS: Newborn screening
NEC: Nasal epithelial cells
PA: Pseudomonas aeruginosa
PI: Pancreatic insufficiency
PS: Pancreatic sufficiency
SCL: Sweat chloride level
SM: Stenotrophomonas malthophilia
